# Physical Activity among Veterans and Nonveterans with Diabetes

**DOI:** 10.1155/2012/135192

**Published:** 2012-07-12

**Authors:** Erin D. Bouldin, Gayle E. Reiber

**Affiliations:** ^1^Health Services Research and Development Service, Department of Veterans Affairs, VA Puget Sound Health Care System, Seattle, WA 98101, USA; ^2^Department of Epidemiology, University of Washington, Seattle, WA 98195, USA; ^3^Department of Health Services, University of Washington, Seattle, WA 98195, USA

## Abstract

Engaging in regular physical activity (PA), with or without a corresponding decrease in weight, is associated with improved health outcomes. The purpose of this study was to quantify the extent to which PA differed between veterans and nonveterans and to determine how diabetes and age influenced this association. Data from the 2009 Behavioral Risk Factor Surveillance System were used in this study. Respondents were classified as having diabetes if they reported ever being diagnosed with diabetes except during pregnancy. Respondents who reported ever serving on active military duty were classified as veterans. Based on self-report, we calculated the average minutes per week of moderate, vigorous, and total activity. After adjusting for sex, race and ethnicity, household income, education level, body mass index (BMI), and recent health checkup, veteran status was associated with a small but significantly larger amount of average weekly moderate PA (2.2 minutes, *P* = 0.0058) but not average weekly vigorous PA (−0.02 minutes, *P* = 0.98). Diabetes and prediabetes were associated with significantly lower mean levels of both moderate and vigorous intensity PA, as was increasing age. Consistent with prior research, veterans engaged in more PA than nonveterans. The association between diabetes, age, and physical activity did not differ by veteran status.

## 1. Introduction

Engaging in regular physical activity, with or without a corresponding decrease in weight, is associated with improved health outcomes among people with diabetes [1–4] and older adults [4–6]. Healthy People 2020 objectives include increasing glycemic, lipid, and blood pressure control among people with diabetes [7], all of which may be facilitated by increased physical activity [1]. Healthy People 2020 also includes an objective to increase the proportion of older adults with functional impairments who engage in leisure-time physical activity [7]. Guidelines from the National Institutes of Health [8] and the American College of Sports Medicine [9, 10] recommend that all adults, including those with diabetes, engage in regular moderate to vigorous physical activity. The 2008 Physical Activity Guidelines for Americans recognizes that any level of physical activity is beneficial, though the evidence is strongest for physical activity at or above the level of the recommended 150 minutes per week of moderate or 75 minutes per week of vigorous activity [4, 8]. They also recognize that older adults may have functional impairments that limit their ability to engage in physical activity, but recommend being as active as possible within safe limits [8]. Although physical activity is increasing among adults in the United States, only half of US adults meet these recommendations [11, 12]. Among older adults, the trend in physical activity is less clear [12], and roughly 40% of adults age 65 and older meet the physical activity guidelines [13].

Veterans in the United States are older, on average, than nonveterans [14] and are more likely than their nonveteran peers to meet physical activity guidelines [15]. Adults with diabetes are less likely to participate in regular exercise and less likely to meet recommendations than their peers without diabetes [16, 17]. Disability status, age, and concurrent health conditions are known to influence physical activity levels among adults [17–19], including among adults with diabetes [2, 18], but it is not clear whether veteran status influences these associations or whether veterans with diabetes and nonveterans with diabetes engage in different levels of physical activity. Therefore, the purpose of this study was to quantify the extent to which physical activity (PA) differed between veterans and nonveterans and to determine how diabetes and age influenced this association.

## 2. Methods

We used data from the Behavioral Risk Factor Surveillance System (BRFSS), a nationally representative cross-sectional telephone survey of noninstitutionalized adults in the United States and its territories coordinated by the Centers for Disease Control and Prevention (CDC), in this study. The BRFSS has demonstrated validity and reliability [20–22]. BRFSS respondents self-report their age. We categorized age into 18–34, 35–44, 45–54, 55–64, 65–74, and 75 and older in order to describe the sample, to calculate mean physical activity by age groups and veteran status, and to assess for effect modification. We used age as a continuous variable in the linear regression models. Respondents were classified as having diabetes if they reported ever being diagnosed with diabetes. Respondents who reported ever being told by their doctor that they have prediabetes or borderline diabetes were classified as having prediabetes. Women with gestational diabetes only and respondents who said they had never been told they have diabetes or pre-diabetes were classified as not having diabetes. 

BRFSS respondents were asked about the amount and frequency of their weekly, nonwork moderate and vigorous intensity physical activity (PA). Moderate activities were defined as causing small increases in breathing or heart rate, while vigorous activities were defined as causing large increases in breathing or heart rate. We calculated the mean minutes of moderate, vigorous, and total (moderate plus vigorous) physical activity per week among respondents and also classified respondents as meeting or not meeting physical activity recommendations according to the 2008 Physical Activity Guidelines for Americans [8]. 

Respondents who reported ever serving on active duty in the military, National Guard, or reserve but who were not currently on active duty at the time of the survey were classified as veterans. Age, gender, educational attainment, race, ethnicity, and annual household income were used as categorical variables. Respondents were classified as having a disability if they reported an activity limitation or reported that they had a health problem requiring the use of special equipment (e.g., cane or wheelchair). We used the question “Would you say that in general your health is excellent, very good, good, fair, or poor?” to assess health-related quality of life and categorized responses into excellent/very good/good and fair/poor. 

All measures used in this study were based on respondent self-report. In 2009, the BRFSS included 432,607 respondents; we excluded 70,469 respondents (16.3%) who were missing data on diabetes status, veteran status, age, sex, race/ethnicity, education, disability, time of most recent health checkup, body mass index (BMI), or average amount of moderate or vigorous physical activity per week. We retained respondents with missing income information but created a separate category for these respondents. Among our final study sample of 362,138, there were 43,048 (8.9%) who reported ever being diagnosed with diabetes, 5,678 who reported ever being diagnosed with pre-diabetes (1.2%), and 48,582 respondents (11.0%) who were veterans. 

To describe the sample by veteran and diabetes status, we calculated proportions and Wald confidence intervals based on the binomial random variables. We used linear regression to model the mean frequency of moderate, vigorous, and total physical activity in an average week in three separate models. We report the  *β*  coefficients from these regression models, which represent the change in minutes of average physical activity per week associated with a one-unit change in the predictor. Because we assessed three physical activity outcomes, we used the Bonferroni correction for multiple comparisons and used an alpha value of 0.0167. We considered sex, race/ethnicity, household income, highest level of education, and body mass index (BMI) to be potential confounders based on previous studies [15–17]. The variables for recent health check-up, health insurance coverage, and having at least one personal health care provider were highly collinear, so we chose the single variable—recent health check-up—that best captured health service utilization since respondents who visited a health care professional may have been advised to engage in regular physical activity. Additionally, we were interested in whether veteran status modified the relationship between diabetes and physical activity or between age and physical activity and used an *a priori* *P*  value of 0.05 to indicate statistical significance of the interaction. Although disability is associated with both diabetes and physical activity [18, 19], we considered disability to be in the causal pathway between diabetes and physical activity and therefore did not adjust for it. With the exception of the physical activity outcomes and age, all variables were categorical.

All analyses were conducted using SAS 9.2 with survey procedures and CDC-assigned weights to account for the complex sampling design of the BRFSS and to represent the US population by age, sex, and race/ethnicity. This study was reviewed as exempt by the VA Puget Sound Health Care System Institutional Review Board.

## 3. Results

Among veterans, the prevalence of diabetes was 15.4% (95% confidence interval [CI]: 14.9, 16.0), and the prevalence of pre-diabetes was 1.8% (95% CI: 1.5, 2.0; Table 1). Among nonveterans, the prevalence of diabetes and pre-diabetes was 8.0% (95% CI: 7.9, 8.2) and 1.2% (95% CI: 1.1, 1.2), respectively. Veterans were older than nonveterans (mean age 59.1 years [SE = 0.2] compared to 44.7 years [SE = 0.1]), and respondents with diabetes were older than respondents without diabetes (mean age with diabetes: 59.3 years [SE = 0.2], mean age with pre-diabetes: 55.6 [SE = 0.5], and mean age with no diabetes: 44.9 years [SE = 0.1]). More than 90% of veterans were male, while 44% of nonveterans were male. The majority of both Veterans and nonveterans reported their race and ethnicity as white, non-Hispanic, though veterans were more likely to be so than nonveterans (78.3% of veterans and 61.7% of nonveterans reported white, non-Hispanic race/ethnicity). Veterans and nonveterans had similar levels of education and annual household income. Disability was significantly more common among veterans: 29.9% of veterans were classified as having a disability compared to 19.4% of nonveterans. Veterans tended to have higher BMI than nonveterans and were more frequently classified as overweight (25.4% of Veterans and 37.4% of nonveterans were classified as ideal weight based on their BMI, and 44.7% of veterans compared to 35.2% of nonveterans were overweight). Veterans were more likely than nonveterans to have any form of health insurance (91.7% versus 84.7%) and less likely to report that cost had been a barrier to accessing health care when needed within the past year (8.3% compared to 15.2%). Veterans also were less likely to report that they did not have a personal doctor (13.3% versus 18.9%) and were more likely to have had a health check-up within the past year (77.4% compared to 67.1%).

The mean amount of moderate physical activity (PA) among veterans was 57.2 minutes per week (standard error [SE] = 0.6), and the mean amount of vigorous physical activity was 35.3 minutes per week (SE = 0.5) for a total mean of 92.5 minutes of physical activity weekly (SE = 0.9; Table 2). The mean minutes of moderate, vigorous, and total PA were 53.3 (SE = 0.3), 33.2 (SE = 0.2), and 86.5 (SE = 0.4) per week, respectively, among nonveterans. Regardless of veteran status, people with diabetes engaged in less moderate PA and vigorous PA per week than people without diabetes. Among veterans with diabetes, the mean moderate, vigorous, and total PA amounts per week were 48.7 (SE = 1.3) minutes, 24.4 (SE = 1.3) minutes, and 73.1 (SE = 2.0) minutes, respectively, while among veterans without diabetes, the means were 59.0 (SE = 0.7) minutes of moderate PA, 37.4 (SE = 0.6) minutes of vigorous PA, and 96.5 (SE = 1.1) minutes of total PA weekly. Veterans with pre-diabetes averaged 48.5 (SE = 3.1) minutes of moderate PA, 29.7 (SE = 2.8) minutes of vigorous PA, and 78.1 (SE = 4.9) minutes of total PA weekly. Among nonveterans with diabetes, mean moderate PA was 42.8 (SE = 0.7) minutes per week, mean vigorous PA was 19.0 (SE = 0.5) minutes per week, and mean total PA was 61.8 (SE = 1.0) minutes per week compared to 54.3 (SE = 0.3) minutes, 34.5 (SE = 0.3) minutes, and 88.8 (SE = 0.5) minutes, respectively, among nonveterans without diabetes. Nonveterans with pre-diabetes averaged 51.3 (SE = 3.0) minutes of moderate PA, 26.4 (SE = 1.7) minutes of vigorous PA, and 77.7 (SE = 4.2) minutes of total PA weekly. Among both veterans and nonveterans, respondents with diabetes had a larger proportion of their total physical activity from moderate physical activity (66.6% of PA among veterans with diabetes, 62.1% of PA among veterans with prediabetes, 69.3% among nonveterans with diabetes, 66.0% among nonveterans with prediabetes, and 61.1% of PA among veterans and nonveterans without diabetes was moderate intensity). Among both veterans and nonveterans, total physical activity decreased with age, with much of the decrease related to a decrease in vigorous activity. Veterans age 18–54 averaged 60.4 (SE = 1.3) minutes of moderate, 42.7 (SE = 1.1) minutes of vigorous, and 103.2 (SE = 2.0) minutes of total PA weekly. Among veterans age 75 and older, the averages were 50.2 (SE = 1.1) minutes of moderate, 24.5 (SE = 0.8) minutes of vigorous, and 74.6 (SE = 1.5) minutes of total PA per week. Nonveterans age 18–54 averaged 55.1 (SE = 0.4) minutes of moderate, 37.3 (SE = 0.3) minutes of vigorous, and 92.4 (SE = 0.6) minutes of total PA weekly. Among nonveterans age 75 and older, the averages were 40.8 (SE = 0.6) minutes of moderate, 13.6 (SE = 0.4) minutes of vigorous, and 54.5 (SE = 0.8) minutes of total PA per week.

When physical activity was categorized based on the Physical Activity Guidelines for Americans recommendations, nearly half of veterans and nonveterans met PA recommendations (48.7% and 49.3%, resp.). Additionally, 37.1% of veterans and 38.3% of nonveterans engaged in some physical activity in an average week but did not meet recommendations, and 14.1% of veterans and 12.3% of nonveterans engaged in no physical activity. 

We adjusted each linear regression model for sex, race and ethnicity, household income, education level, BMI category, and recent health check-up. In the fully adjusted models (Table 3), we found no evidence of effect modification by veteran status (*P* = 0.07  for diabetes and  *P* = 0.89  for age in categories) so report results adjusted by veteran status rather than stratified.

In the adjusted moderate PA model, veterans engaged in a small but statistically significantly higher amount of PA on average (2.2 minutes; 95% CI: 0.6, 3.8;  *P* = 0.0058). Respondents with diabetes averaged 8.0 (95% CI: −9.4, −6.5;  *P* < 0.0001) fewer minutes, and respondents with pre-diabetes averaged 1.3 fewer minutes, (95% CI: −6.3, 3.7;  *P* = 0.62) of moderate PA weekly than respondents without diabetes after adjusting for covariates. For each one year increase in age, respondents averaged 0.1 fewer minutes (95% CI: −0.2, −0.1;  *P* < 0.001) of moderate PA weekly, adjusted for covariates.

In the adjusted vigorous PA model, there was no statistically significant difference in average weekly PA by veteran status (−0.02 minutes; 95% CI: −1.3, 1.3;  *P* = 0.98). Respondents with diabetes averaged 7.2 (95% CI: −8.3, −6.1;  *P* < 0.0001) fewer minutes and respondents with pre-diabetes averaged 1.5 fewer minutes (95% CI: −4.4, 1.3;  *P* = 0.30) of vigorous PA weekly than respondents without diabetes after adjusting for covariates. For each one year increase in age, respondents averaged 0.3 fewer minutes (95% CI: −0.4, −0.3;  *P* < 0.001) of vigorous PA weekly, adjusted for covariates.

In both models, women averaged significantly less weekly PA than men (data not shown; available upon request). Respondents who reported Hispanic ethnicity had a small but statistically significantly lower mean amount of moderate PA weekly compared to white, non-Hispanic respondents. For other categories of race and ethnicity, the trend was not statistically significant in either model. People with lower levels of education had significantly higher weekly PA than people with a college degree or higher, on average. The relationship between income and PA differed by the type of PA: for moderate PA, people with lower income tended to have higher mean PA, while for vigorous PA, people with lower income had a higher mean PA. Across both moderate PA and vigorous PA models, overweight and obesity were associated with a significantly lower amount of PA per week, on average. 

## 4. Discussion

We identified a strong and statistically significant association between diabetes and physical activity and between age and physical activity among both veterans and nonveterans. The association between pre-diabetes and physical activity was much weaker. The demographic profile of veterans in this study was similar to a previous analysis of BRFSS data and VA records [23]. Consistent with the limited prior research on the topic [15], veterans engaged in more physical activity than their nonveteran peers. As suggested by Littman et al. [15], this may be a result of their fitness for military service or a result of the tendency to continue physical activity after the high level of activity associated with military training and service. Factors associated with lower physical activity were consistent across veterans and nonveterans.

We classified respondents who had been told by a doctor that they had pre-diabetes or borderline diabetes as a separate category in this study. These individuals likely had impaired fasting glucose or glucose tolerance or high hemoglobin A1c [24] and are therefore at higher risk for developing diabetes and for poor cardiovascular health [24, 25]. In this study, respondents with pre-diabetes had, on average, higher levels of physical activity and lower levels of disability than respondents with full-blown diabetes; including these respondents in the group with diabetes would have attenuated differences between respondents with and without diabetes. Diabetes and pre-diabetes are known to be underdiagnosed: according to an analysis of National Health and Nutrition Examination Survey (NHANES) data by Cowie et al. [26], 19.0% of adults had undiagnosed diabetes, and an additional 3.5% were at high risk for diabetes based on their hemoglobin A1c. In a BRFSS reliability study, Martin and colleagues found that the sensitivity of BRFSS diabetes question had moderate sensitivity and high specificity compared to medical record review [22]. The underdiagnosis of diabetes and pre-diabetes and the moderate sensitivity of the diabetes question used in this study likely led to misclassification in this study which may have resulted in underestimating the association between diabetes or pre-diabetes and physical activity.

Data from the BRFSS are designed to represent the noninstitutionalized adult population; therefore, these results are generalizable to the community-dwelling residents of the United States and its territories who age 18 and older. These data are cross-sectional, so the temporal sequence of the associations reported is unclear. It may not be true that veteran status or diabetes causes people to be more or less physically active. Nonetheless, there is a public health benefit to quantifying the amount of PA among people with diabetes, and the relationship between diabetes, pre-diabetes, and physical activity identified in this study may be useful to health care providers advising patients. Likewise, because of the cross-sectional nature of the data, we cannot be sure from this study that aging causes a decline in physical activity since individuals were not followed over time. However, given the body of existing research on the topic, we feel the decline in activity and the capacity for exercise associated with aging has been well established [4, 27, 28]. 

All data in this study were self-reported and therefore are subject to recall and social desirability biases. Based on data from the 2003-2004 NHANES, in which height and weight were measured directly and accelerometers were used to capture physical activity information, the proportion of US adults who meet physical activity guidelines was below 5% [29], much lower than the 50% estimate in this study and others (e.g., [15, 17]) obtained from self-reported data. This overreporting may result in nondifferential misclassification (if all respondents overreported their physical activity). It is possible that a differential social desirability bias exists since respondents with diabetes may have received physician advice to exercise more often than adults without diabetes. For these reasons, the difference in estimates between groups in this study may be more appropriate than treating the means or proportions themselves as accurate reflections of physical activity among veterans and people with diabetes in the general population. 

Although the estimated differences in physical activity minutes per week were small, they are meaningful in terms of health outcomes. For example, Buman et al. [30] found that replacing 30 minutes of sedentary behavior each day with light intensity physical activity was associated with improved physical health among older adults; this benefit increased as the intensity of activity increased. Likewise, Healy et al. [31] found that light intensity physical activity was associated with improved glucose control, as measured by the two-hour postchallenge plasma glucose but not fasting glucose. Additional research is needed to understand whether self-reported moderate physical activity includes light intensity physical activity, particularly among older adults [32], including veterans and individuals with diabetes [33]. Such research may include an assessment of whether additional categories of physical activity should be routinely added to health surveys like the BRFSS. 

## Figures and Tables

**Table 1:** Demographic and health characteristics of veterans and nonveterans in the United States, 2009 Behavioral Risk Factor Surveillance System.

Variable	Categories	Veterans	Nonveterans
		weighted % (95% CI)	weighted % (95% CI)
	Diabetes	15.4 (14.9, 16.0)	8.0 (7.9, 8.2)
Diabetes diagnosis	Pre-diabetes	1.8 (1.5, 2.0)	1.2 (1.1, 1.2)
	No diabetes	82.8 (82.1, 83.4)	90.8 (90.6, 91.0)

Age	18–34	9.5 (8.7, 10.3)	32.5 (32.1, 32.9)
	35–44	12.4 (11.7, 13.1)	20.1 (19.8, 20.4)
	45–54	15.2 (14.6, 15.8)	19.9 (19.7, 20.2)
	55–64	23.2 (22.5, 23.8)	13.7 (13.5, 13.9)
	65–74	19.0 (18.5, 19.6)	7.7 (7.6, 7.9)
	75+	20.7 (20.1, 21.2)	5.9 (5.8, 6.0)

Sex	Male	91.8 (91.3, 92.3)	44.4 (44.0, 44.8)

Race, ethnicity	White, non-Hispanic	78.3 (77.4, 79.2)	61.7 (68.5, 69.3)
	Black, non-Hispanic	9.7 (9.0, 10.3)	9.7 (9.5, 9.9)
	Other race, non-Hispanic	5.2 (4.7, 5.6)	6.7 (6.5, 6.9)
	Any race, Hispanic	6.8 (6.1, 7.5)	14.7 (14.3, 15.0)

Highest level of education	Less than high school	5.0 (4.6, 5.3)	10.2 (10.0, 10.5)
	High school degree or equivalent	28.3 (27.5, 29.1)	27.4 (27.1, 27.7)
	Some college	31.2 (30.3, 32.0)	26.5 (26.2, 26.8)
	College or beyond	35.5 (34.7, 36.3)	35.8 (35.5, 36.2)

Annual household income	<$15,000	4.9 (4.5, 5.2)	9.2 (9.0, 9.5)
	$15,000–$24,999	13.2 (12.6, 13.8)	13.7 (13.5, 14.0)
	$25,000–$49,999	26.9 (26.1, 27.6)	21.7 (21.4, 22.0)
	$50,000–$74,999	17.7 (17.1, 18.4)	14.4 (14.1, 14.7)
	≥$75,000	28.6 (27.8, 29.4)	30.2 (29.8, 30.5)
	Missing	8.9 (8.2, 9.2)	10.8 (10.5, 11.0)

Disability status	Disability	29.9 (29.1, 30.7)	19.4 (19.2, 19.7)

General health rating	Fair or poor	19.2 (18.5, 19.8)	14.9 (14.6, 15.1)

Body mass index (BMI)	Ideal (<25.0)	25.4 (24.6, 26.2)	37.4 (37.1, 37.8)
	Overweight (25.0–29.9)	44.7 (43.8, 45.5)	35.2 (34.8, 35.5)
	Obese (≥30.0)	29.9 (29.1, 30.7)	27.4 (27.0, 27.7)

Health insurance coverage	Any (including government/VA)	91.7 (91.0, 92.3)	84.7 (84.3, 85.0)

Health care cost	Time in past 12 months when could not see doctor because of cost	8.3 (7.7, 8.9)	15.2 (14.9, 15.5)

Health provider	No personal doctor	13.3 (12.5, 14.0)	18.9 (18.6, 19.3)

Last health check-up	Within past year	77.4 (76.6, 78.2)	67.1 (66.8, 67.5)
	1–5 years ago	16.4 (15.7, 17.1)	23.6 (23.2, 23.9)
	Never	6.2 (5.8, 6.6)	9.3 (9.0, 9.5)

CI: confidence interval.

**Table 2:** Mean minutes of moderate, vigorous, and total physical activity in an average week reported by veterans and nonveterans with and without diabetes in the United States, 2009 Behavioral Risk Factor Surveillance System.

Demographic group	Moderate	Vigorous	Total
	mean (SE)	mean (SE)	mean (SE)
Veterans (*n* = 48,582)	57.2 (0.6)	35.3 (0.5)	92.5 (0.9)
With diabetes (*n* = 8,515)	48.7 (1.3)	24.4 (1.3)	73.1 (2.0)
With pre-diabetes (*n* = 947)	48.5 (3.1)	29.7 (2.8)	78.1 (4.9)
Without diabetes (*n* = 39,120)	59.0 (0.7)	37.4 (0.6)	96.5 (1.1)
Age 18–54 (*n* = 10,471)	60.4 (1.3)	42.7 (1.1)	103.2 (2.0)
Age 55–64 (*n* = 12,664)	57.5 (1.0)	36.1 (1.1)	93.6 (1.7)
Age 65–74 (*n* = 12,507)	58.3 (1.0)	31.5 (0.8)	89.9 (1.5)
Age ≥ 75 (*n* = 12,940)	50.2 (1.1)	24.5 (0.8)	74.6 (1.5)
Nonveterans (*n* = 313,556)	53.3 (0.3)	33.2 (0.2)	86.5 (0.4)
With diabetes (*n* = 34,533)	42.8 (0.7)	19.0 (0.5)	61.8 (1.0)
With pre-diabetes (*n* = 4,731)	51.3 (3.0)	26.4 (1.7)	77.7 (4.2)
Without diabetes (*n* = 274,292)	54.3 (0.3)	34.5 (0.3)	88.8 (0.5)
Age 18–54 (*n* = 158,990)	55.1 (0.4)	37.3 (0.3)	92.4 (0.6)
Age 55–64 (*n* = 68,919)	50.6 (0.5)	26.4 (0.4)	76.9 (0.7)
Age 65–74 (*n* = 49,040)	50.8 (0.5)	21.9 (0.4)	72.7 (0.7)
Age ≥ 75 (*n* = 36,607)	40.8 (0.6)	13.6 (0.4)	54.5 (0.8)

SE: standard error.

**Table 3:** Adjusted^∗^ estimated differences in mean minutes of moderate, vigorous, and total physical activity (PA) per week among veterans and nonveterans in the United States, 2009 Behavioral Risk Factor Surveillance System.

Variable	Category	Moderate PA	Vigorous PA	Total^†^ PA
		minutes (**β**) (95% CI)	minutes (**β**) (95% CI)	minutes (**β**) (95% CI)
Veteran status	Veteran	2.2 (0.6, 3.8)	−0.02 (−1.3, 1.3)	2.2 (−0.1, 4.5)
	Nonveteran	Reference	Reference	Reference

Diagnosed diabetes status	Diabetes	−8.0 (−9.4, −6.5)	−7.2 (−8.3, −6.1)	−15.2 (−17.3, −13.1)
	Pre-diabetes	−1.3 (−6.3, 3.7)	−1.5 (−4.4, 1.3)	−2.8 (−9.8, 4.2)
	Neither diabetes nor pre-diabetes	Reference	Reference	Reference

Age	One-year increase	−0.1 (−0.2, −0.1)	−0.3 (−0.4, −0.3)	−0.5 (−0.5, −0.4)

*Adjusted for sex, race/ethnicity, education, household income, body mass index, and last health check-up.

^†^Total physical activity is the sum of moderate and vigorous physical activity.

**β**: linear regression coefficient, indicating the minutes per week associated with a one-unit change in the exposure variable.

CI: confidence interval.
